# Lacrimal Gland Repair after Short-term Obstruction of Excretory Duct in Rabbits

**DOI:** 10.1038/s41598-017-08197-2

**Published:** 2017-08-15

**Authors:** Hui Lin, Ying Liu, Hong He, Benjamin Botsford, Samuel Yiu

**Affiliations:** 1 Wilmer Eye Institute, School of Medicine, Johns Hopkins University, Baltimore, MD United States; 2 Tufts University School of Medicine, Boston, MA United States

## Abstract

Aqueous tear-deficient dry eye is a multifactorial chronic disorder in which the lacrimal glands fail to produce enough tears to maintain a healthy ocular surface. The existence of lacrimal gland stem/progenitor cells was proposed in several species, yet their origin and characteristics are not very clear. Here, we investigated the presence of resident progenitor cells and their regenerative potential in a rabbit model with lacrimal gland main excretory duct ligation-induced injury. The ligation-injured lacrimal glands temporarily decreased in weight and had impaired tear secretion. Protein expression profiles and transcriptional profiles were obtained from injured tissue. Isolated lacrimal gland progenitor cells were tested and characterized by stem cell-related marker evaluation, single cell clonal assay and three-dimensional (3-D) culture. The results of our study indicate that lacrimal glands are capable of tissue repair after duct ligation-induced injury, likely involving resident stem/progenitor cells and epithelial-mesenchymal transitions. Lacrimal gland progenitor cells isolated from ligated tissue can differentiate in 3-D culture. The results provide further insights into lacrimal gland stem/progenitor cell physiology and their potential for treating severe cases of tear deficiency.

## Introduction

Dry eye syndrome is a multifactorial disease that results in symptoms of discomfort, visual disturbance, and tear film instability with potential to damage the ocular surface and even lead to blindness. It is one of the most common ocular disorders in the United States, with approximately 4.91 million Americans affected by the disease^1^. Aqueous tear-deficient dry eye is the most common form of severe dry eye syndrome, where lacrimal glands fail to produce sufficient tears to maintain the integrity of the tear film and a healthy ocular surface. Current treatment modalities, including intensive artificial tear supplements, punctal occlusion, bandage contact lenses, use of anti-inflammatory medications or pharmacological stimulation of tear secretion, are palliative and conservative^1, 2^. Although these approaches may provide temporary symptomatic relief, they do not address the underlying lacrimal gland damage process. A recently reseach showed a therapeutic effect of defined mouse lacrimal gland progenitor cell transplantation in lacrimal gland dysfunction models^3^. Thus, there is a need to thoughly investigate the lacrimal gland progenitor cells characteristics for better development of cell therapy for severe aqueous tear-deficient dry eye.

Stem/progenitor cells in adult tissues have been extensively studied because they are capable of self-renewal and differentiation and have potentially wide-ranging clinical use. In contrast to the large literature on stem/progenitor cells in the pancreas, salivary glands, and mammary glands^4–7^, there have been relatively few studies addressing the lacrimal glands, and these have employed only rodent models^8–10^.

Duct ligation-induced injury was used in several gland tissues to study the regenerative process and suggested the proliferation of duct epithelial cells plays a critical role in the initiation of gland regeneration. The studies on salivary gland^11–14^, pancreas^15–17^, liver^18^, intestine^19^, and mammary glands^4^ reported self-regenerating capabilities of these tissues. In the salivary gland duct ligation model, the proliferation of different cell types including acinar, ductal, and/or myoepithelial cells accompanies tissue repair after ligature releasing^20–23^. Although similar studies on the lacrimal gland are still lacking, it was reported that the murine lacrimal gland is capable of self-repair following experimentally induced injury by injection of interleukin-1 (IL-1) into the exorbital lacrimal glands^24, 25^.

In terms of the cell source for tissue repair and regeneration, one theory advocates expansion of stem/progenitor cells^17, 23^, and another advocates the trans-differentiation/dedifferentiation-rediffentiation process^26^. It is difficult to distinguish between these two hypothesis during tissue repair in most mammalian species. In aid of genetic approaches for lineage tracing, the origin of the regenerated cells, might be demonstrated, but the results remain controversial, especially for the pancreas^16^. Meanwhile, some studies isolated and expanded stem/progenitor cells from the salivary glands and the pancreas to support the expanaion theory^27–29^. Although the issue of stem/progenitor cells versus trans-differentiation is still hotly debated, it seems most likely that more than one mechanism may apply in tissue repair.

In the current study, our team developed a method of temporarily ligating the main excretory duct of the rabbit lacrimal gland and examined the subsequent effects. Lacrimal gland progenitor cells were cultured to show their potential regenerative effect during tissue repair.

## Results

### Changes in Gland Weights and Tear Secretion After Duct Ligation Injury

**Figure 1:**
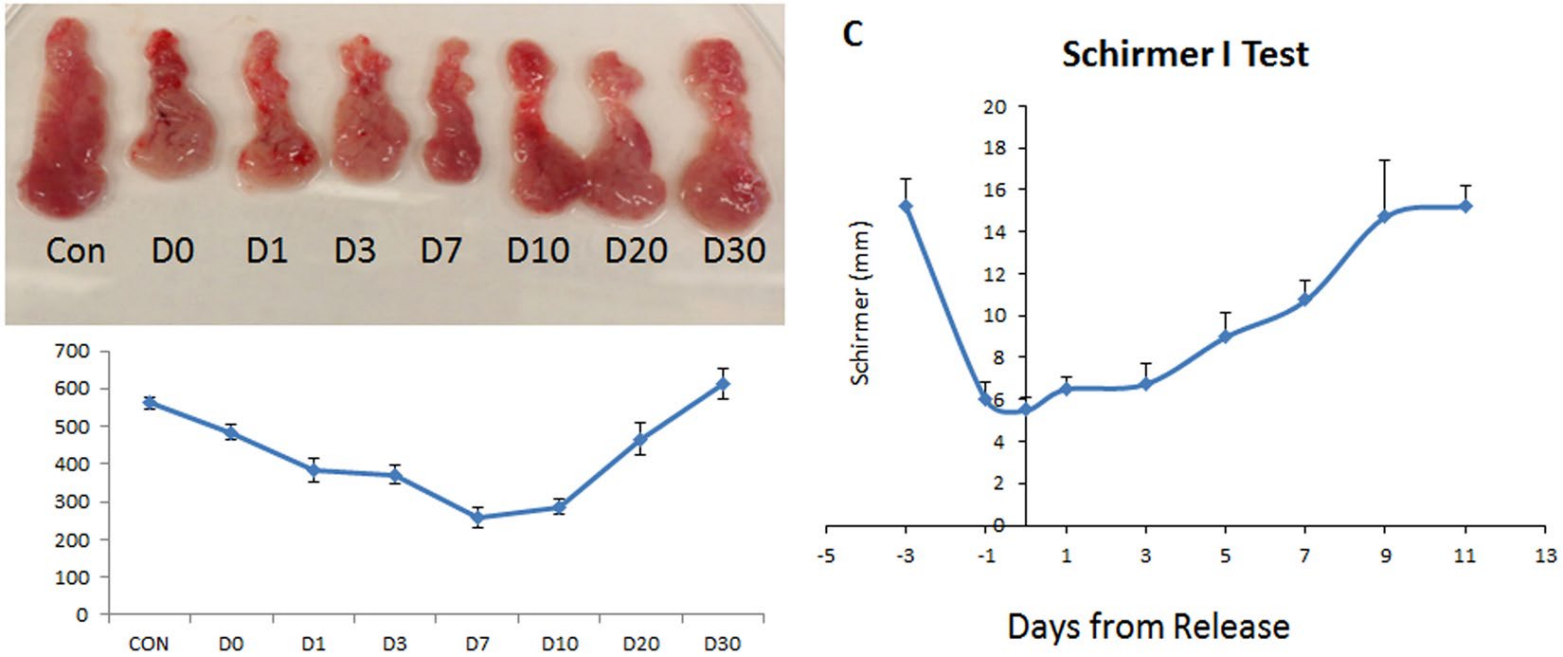
Gross morphology, weight and tear secretory function after ligation-induced injury. The size and weight of rabbit lacrimal glands decreased after reopening of the lacrimal gland main execretory duct from days 0–7 and then gradually recovered from days 10–30 (**A**,**B**). The Schirmer test showed a considerable decrease in tear secretion after ligation; it gradually increased to baseline within 10 days after release of the ligature (**C**). Con = control, D = day.

In the early stage of reopening after ligation-induced injury, the size and weight of rabbit lacrimal gland tissues decreased and then gradually recovered (see, Fig. 1A,B). The Schirmer test, which assays tear quantity, showed comparable changes (Fig. 1C). These results indicate that ligation of the main excretory duct of the rabbit lacrimal gland for three days led to decreased tear secretion and atrophy of the gland. Reduction of tear secretion was reversible after reopening the main excretory duct. Similar recovery in total gland weight followed reopening, albeit lagged behind tear secretion recovery.

### Proliferation of Lacrimal Gland Epithelial Cells After Duct Ligation-Induced Injury

**Figure 2:**
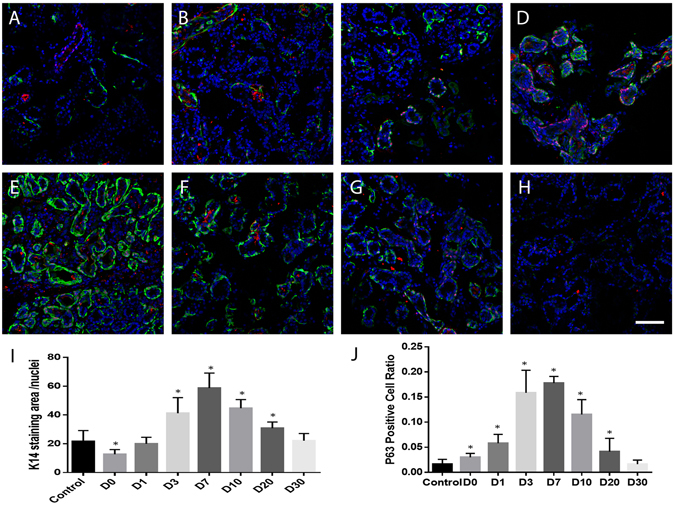
K14 and ∆Np63 double staining of lacrimal glands after ligation-induced injury. Immunofluorescence staining of K14 (green) and ∆Np63 (red) on control lacrimal gland (**A**), and samples 0 (**B**), 1 (**C**), 3 (**D**), 7 (**E**), 10 (**F**), 20 (**G**) and 30 (**H**) days post-reopening of the main excretory duct. K14 expression pattern (**A**–**H**) and level (**I**) changed after ligation-induced injury. ∆Np63 positive cell number (**J**) is significantly higher in D0 to D20 samples. Blue is DAPI staining. D = Day. *P < 0.05. Bar represents 100 μm.

Presence of K14 keratin, a basal epithelial cell marker that is reported to be confined to the basal cell compartment in normal ocular surface epithelium, indicates high cellular proliferative capability^30^. It is also believed to be one of potential lacrimal gland progenitor cell markers^31, 32^. Immunohistochemical analysis demonstrated that, in contrast to the control group (Fig. 2A, green), which has scattered K14 expression in the myoepithelial cell location, ligated tissues, especially at 3 days (Fig. 2D), 7 days (Fig. 2E), and 10 days (Fig. 2F) after reopening expressed significantly higher amount of K14-positive cells in the cell clusters and ductlike structures (Fig. 2I). Meanwhile, staining of ∆Np63–the ocular surface epithelial cell proliferation marker^8^–showed positive nuclear staining only in the relatively large duct lumen in normal lacrimal gland tissue (Fig. 2A, red). However, ligation group samples have significantly higher amounts of positive nuclear staining of ∆Np63 cells in the ductlike structures (Fig. 2J).

**Figure 3:**
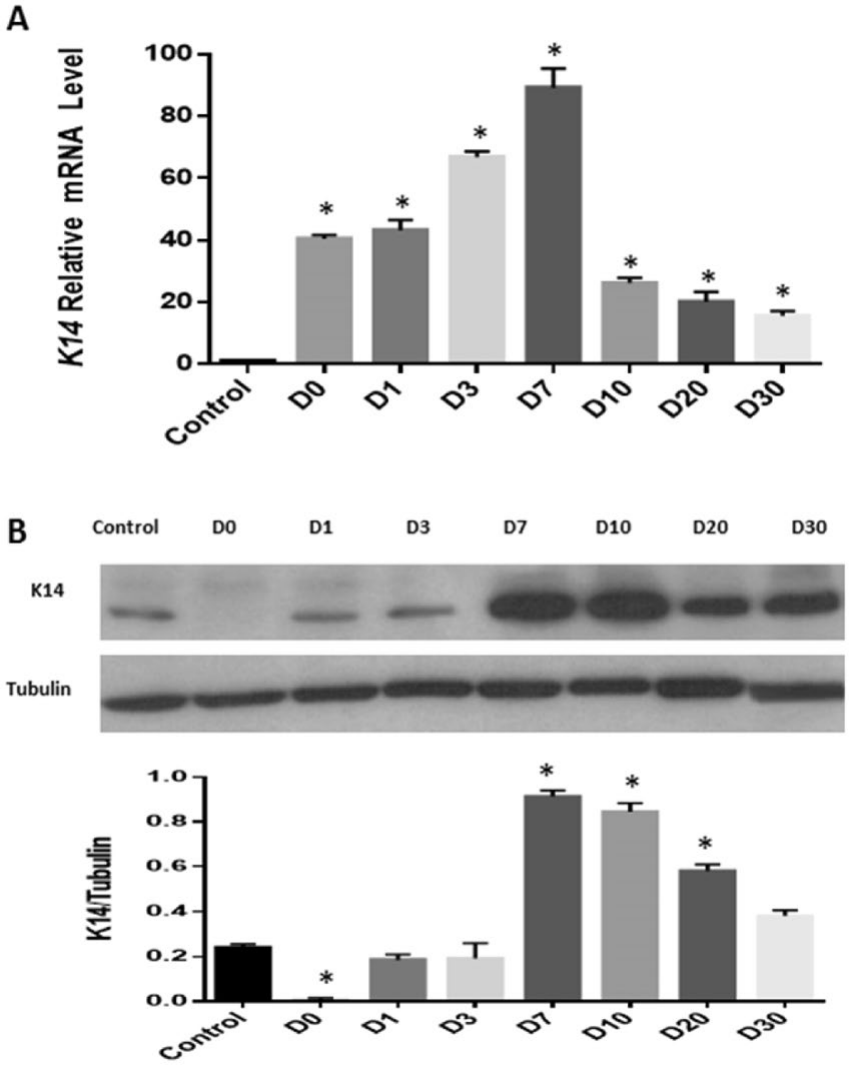
K14 level changes in lacrimal glands after ligation-induced injury. RT-qPCR showed that *K14* gene expression dramatically increased after main excretory duct reopening on days 0–30 ((**A)** *P < 0.05). Western blot analysis (**B**) demonstrated K14 expression level change in tissues post-ligation opening.

As shown in Fig. 3A, RT-qPCR analysis demonstrated that K14 mRNA levels were up-regulated after duct reopening. Immunoblotting also showed significant level changes of K14 in ligation samples (Fig. 3B). Because the peak of K14 mRNA and protein expression was on day 7 after reopening, we chose day 7 as the timepoint for more-detailed analysis of epithelial markers and a cell isolation study.

**Figure 4:**
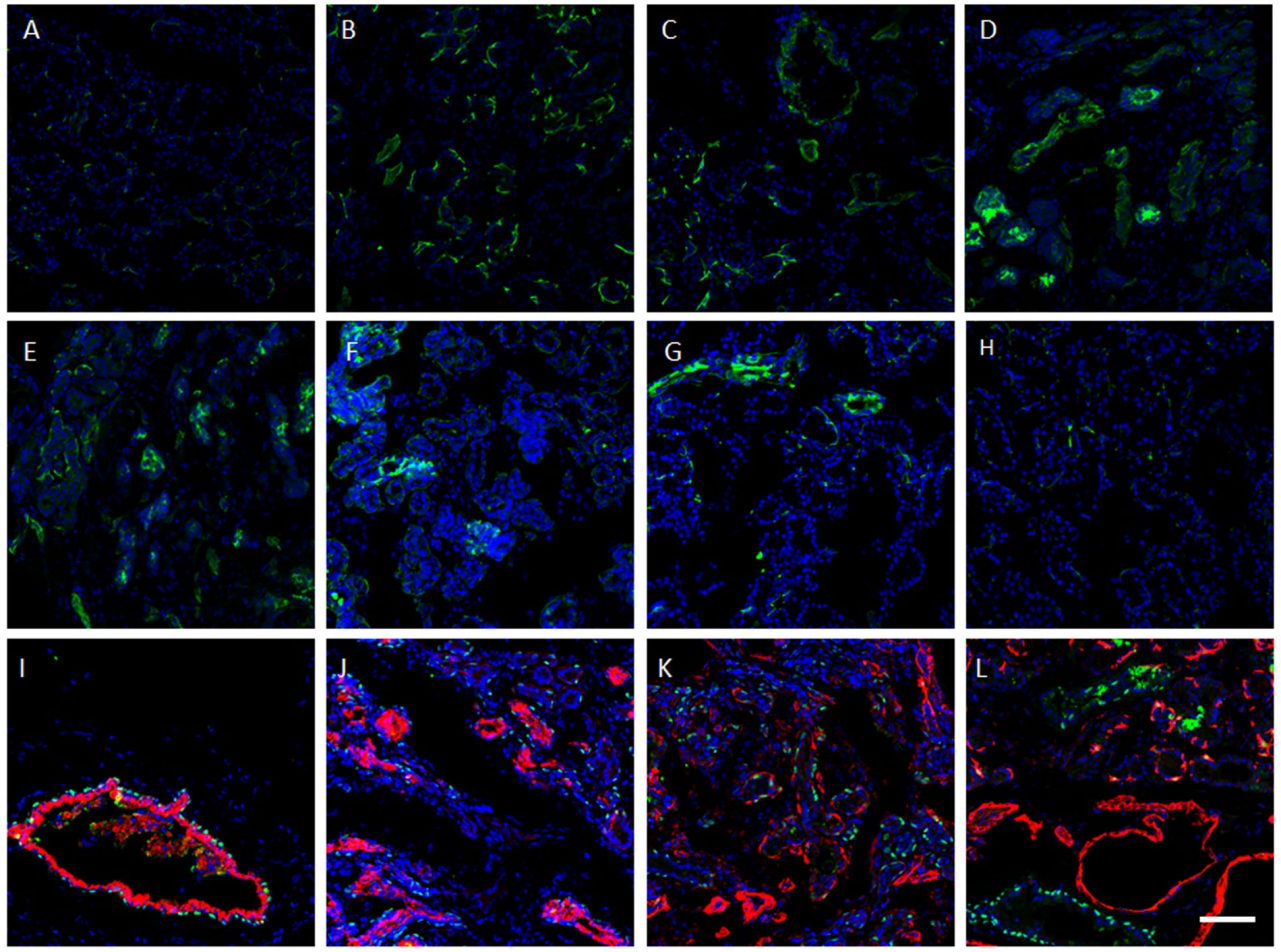
Immunostaining in lacrimal glands after ligation-induced injury. Immunofluorescence staining of nestin (**A**–**H**, green); ∆Np63 (**I**–**J**, green); K4 (**I**,**J**, red) and SMA (**K**,**L**, red) on control lacrimal gland (**A**), and samples 0 (**B**), 1 (**C**), 3 (**D**), 7 (**E**,**I**–**L**), 10 (**F**), 20 (**G**) and 30 (**H**) days post-reopening of the main excretory duct. Blue is DAPI staining. Bar represents 100 μm.

To identify the origin of those proliferated cells, immunofluorescence staining of nestin, keratin 4 (K4), and alpha-smooth muscle actin (SMA) were performed (Fig. 4). Nestin (a progenitor cell marker) staining showed a similar pattern to that of K14 staining, which is positive only in some scattered myoepithelial cell locations in control samples, but has more abundant positive staining in small cell cluster and ductlike structures in ligation group samples. The double staining of ∆Np63 and K4 (lacrimal gland duct cell marker)^32^ and ∆Np63 and SMA (myoepithelial marker)^8^ showed that only partial ∆Np63 positive proliferation cells are positive for K4 (Fig. 4J) or SMA (Fig. 4K). The epithelium in the large duct lumen is positive for ∆Np63 and K4 (Fig. 4I), but not SMA (Fig. 4L).

**Figure 5:**
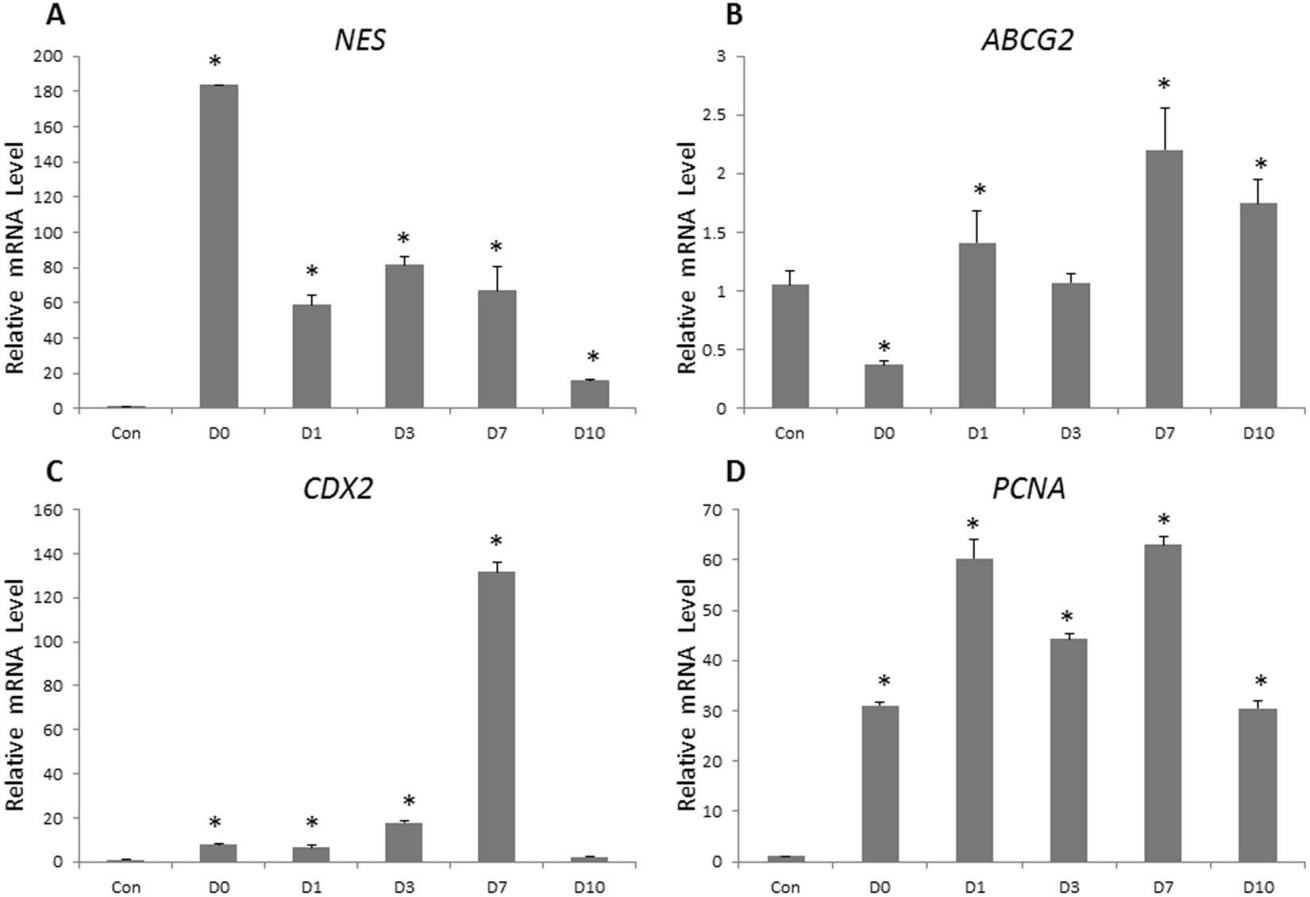
Epithelial proliferation-associated gene expression levels change in lacrimal gland tissues after duct ligation-induced injury. Real-time quantitative reverse transcription PCR (RT-qPCR) showed that *NES* (**A**), *ABCG2* (**B**), *CDX2* (**C**), and *PCNA* (**D**) gene expression dramatically increased after the main excretory duct ligation and reopening process (*P < 0.05).

RT-qPCR results also demonstrated that mRNA levels of stem cell-related or proliferation-associated genes *NES, ABCG2, CDX2*, and *PCNA* were significantly up-regulated in ligated then opened samples when compared to control samples (Fig. 5).

Collectively, these results indicate that lacrimal gland repair after duct ligation-induced injury is associated with the active proliferation of lacrimal gland epithelial cells after ligature release. However, the emergence of those proliferating cells might involve more than one mechanism.

### The Epithelial-Mesenchymal Transition (EMT) Occurred in Lacrimal Glands After Duct Ligation-Induced Injury

**Figure 6:**
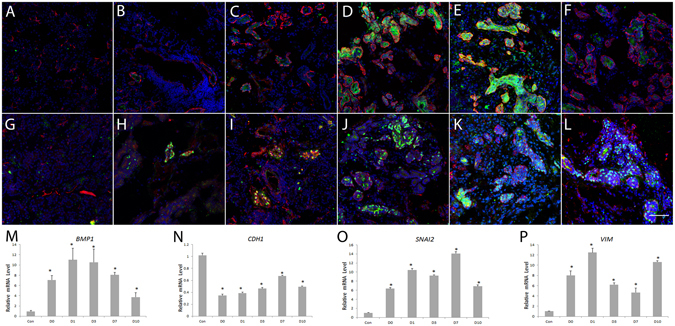
EMT-associated markers in lacrimal gland tissues after duct ligation-induced injury and ligature release. Double staining on control lacrimal gland (**A**,**G**), and samples 0 (**B**,**H**), 1 (**C**,**I**), 3 (**D**,**J**), 7 (**E**,**K**) and 10 **(F**,**L**) days post-reopening of the main excretory duct showed colocalizaion of Vimentin (red, **A**–**L**) with epithelia proliferation markers K14 (green, **A**–**F**) and ∆Np63 (green, **G**–**L**) during tissue repair. Blue is DAPI staining. Bar represents 100 μm. RT-qPCR showed that *BMP1* (**A**), *CDH1* (**B**), *SNAI2* (**C**), and *VIM* (**D**) mRNA levels dramatically changed after main secretory duct ligation and reopening (*P < 0.05).

The EMT is a process by which epithelial cells lose their cell polarity and cell-cell adhesion, and gain migratory and invasive properties to become mesenchymal stem cell-like cells. The EMT is essential for tissue repair and regeneration. To examine the possibility of EMT process in those proliferated cells, we performed double staining of EMT related marker Vimentin and epithelial cell proliferation markers (K14 and ∆Np63) during tissue repair. The colocolizaion of Vimentin and K14/ ∆Np63 was found in small cell cluster and ductlike structures in ligation group samples (Fig. 6A–L), especially on D3 and D7. We also examined the EMT-related genes *BMP1*, *CDH1*, *VIM*, and *SNAI2*. Analysis by RT-qPCR (Fig. 6M–P). showed that *BMP1*, *SNAI2*, and *VIM* were significantly up-regulated in the ligation and release group compared to controls. On the other hand, expression of *CDH1* (E-cadherin) was dramatically down-regulated in the ligation samples.

### Characterization of Proliferative Lacrimal Gland Epithelial Cells Isolated from Lacrimal Tissues with Duct Ligation-Induced Injury and Ligature Release

To examine the proliferative capability and characterization of the lacrimal gland epithelial cells isolated from the injured tissues, we characterized lacrimal gland samples after main excretory duct ligation and reopening via cell isolation and culture. Data are from day 7 post-ligature opening samples.

**Figure 7:**
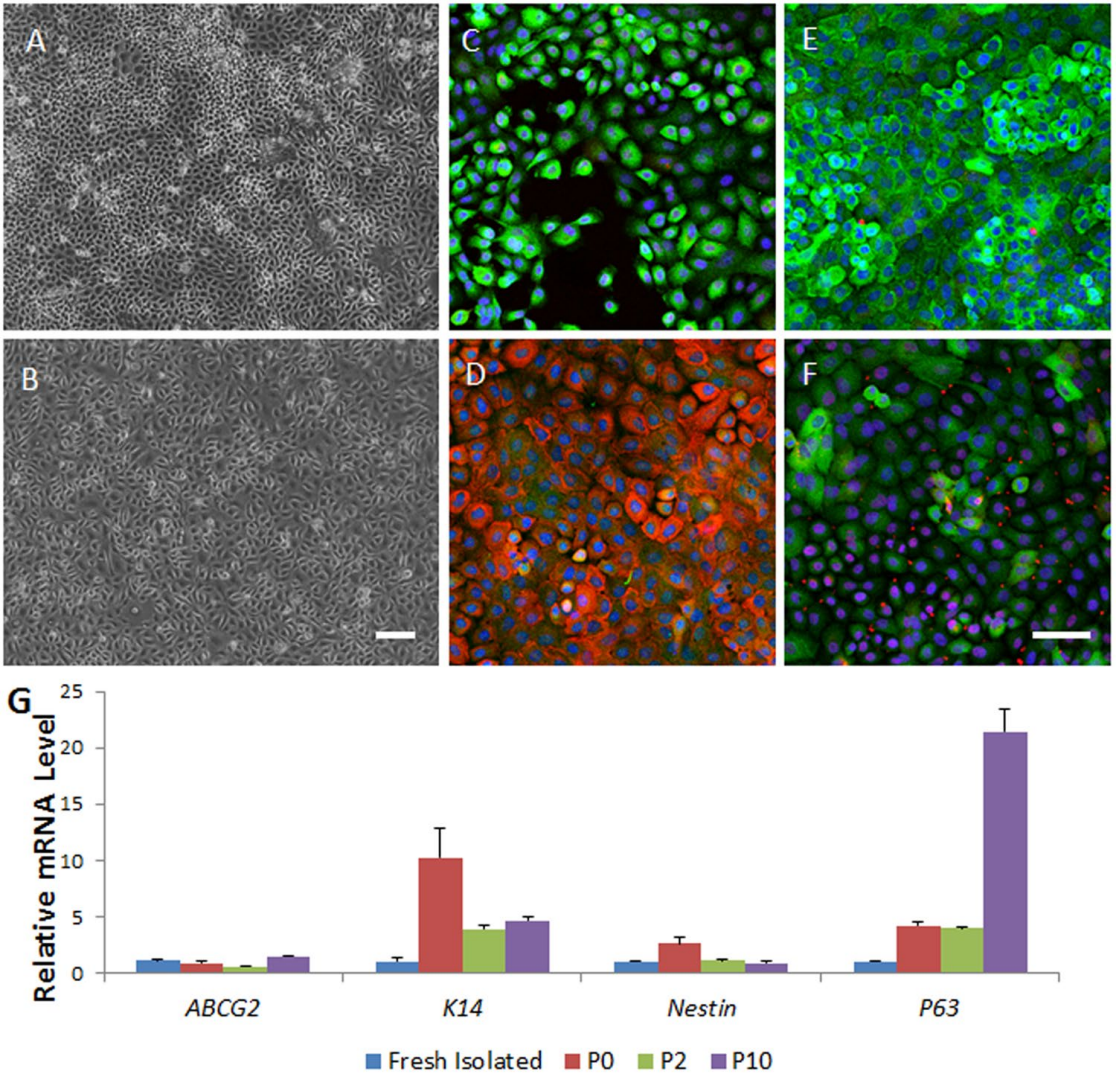
The proliferative capability and characterization of the immature lacrimal gland epithelial cells in monolayer cultured in serum-free media. Monolayer culture of cells from day 7 samples after duct ligature release can form a compactly organized epithelial morphology from passage 0 (P0, **A**) to P12 (**B**). Immunostaining showed most of the cells to be nestin positive (green, **C**), ∆Np63 positive (red, **C**,**F**), ABCG2 positive (red, **D**), PCK positive (green, **D**) and K15 positive (green, E). Some of these P10 cells to be K14 positive (green, **F**), RT-qPCR showed the mRNA level of progenitor cell-related genes in the cultured cells (**G**). Bar represents 100 μm.

The single clonal assay showed that nearly 1% of the expanded lacrimal gland epithelial cells in passage 1 (P1) from the day 7 post-ligature group could form a confluent monolayer and then be continuously passaged to at least P12 (Fig. 7A,B). These proliferative lacrimal gland epithelial cells expressed progenitor cell markers Nestin (Fig. 7C, green), ABCG2 (Fig. 7D, red), ∆Np63 (Fig. 7C,F, red), K15 (Fig. 7E, green)and K14 (Fig. 7F, green), and are epithelial marker pan-cytokeratin (PCK) (Fig. 7D, green) positive and myoepithelial marker SMA (Fig. 7E, red) negative in P10. RT-qPCR also showed that the cells maintained high levels of expression of the proliferation-related genes *K14* and *P63* from P0 to P10 (Fig. 7G).

### Differentiation Potential of Lacrimal Gland Epithelial Cells in 3-D Culture

**Figure 8:**
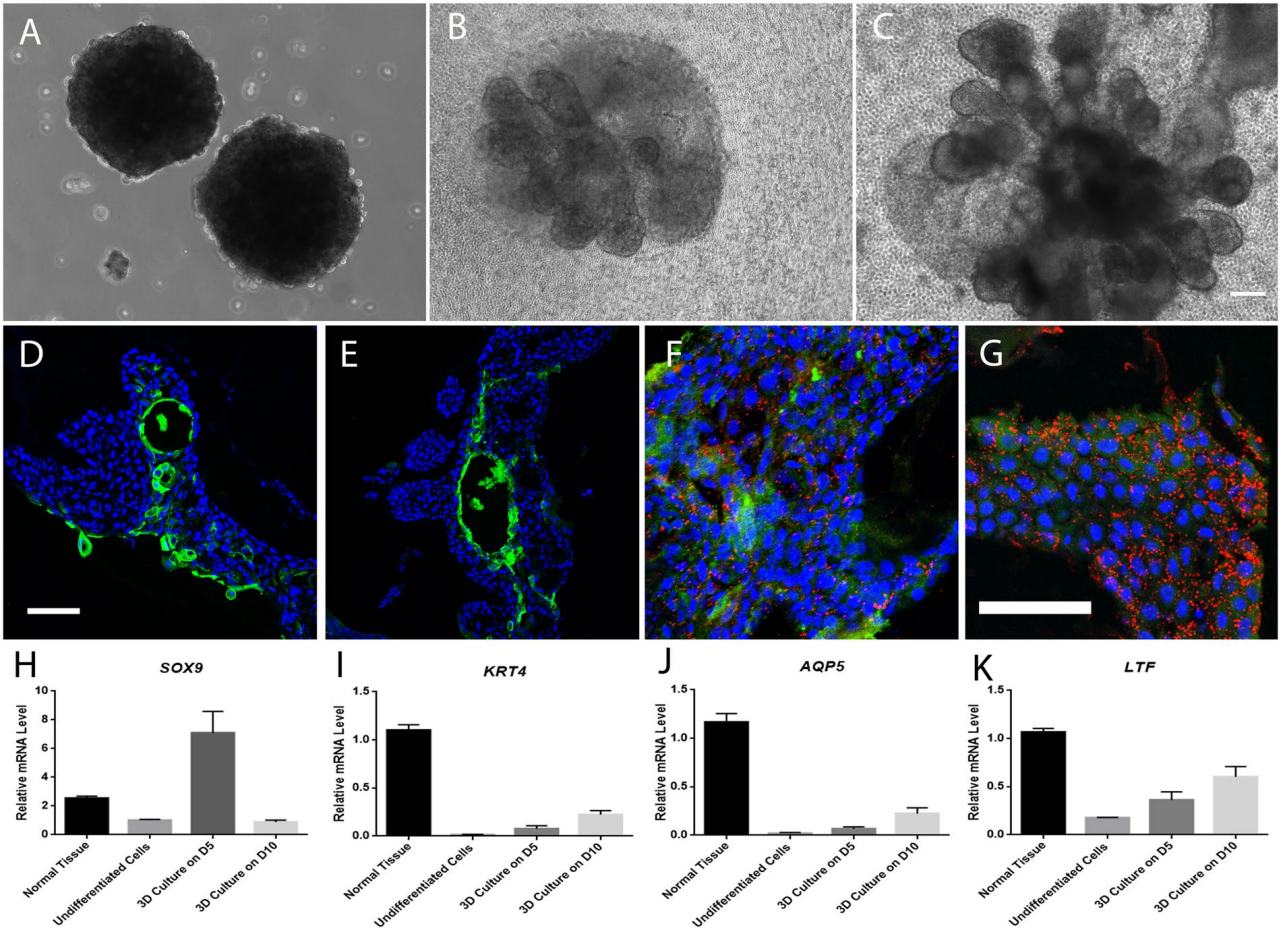
3-D culture of lacrimal gland spheres. Lacrimal gland epithelial cells from day 7 post-reopening in P2 formed similarly sized lacrimal spheres in suspension culture (**A**), and gradually branched out in about 2 weeks (days in culture, 5, (**B**); 10, (**C**)) to form mini gland-like structure in laminin gel. Immunostaining demonstrated K4 (green, **D**,**E**) positive, Lactoferrin (red, **F**,**G**) positive and AQP5 positive (green, **F**,**G**) cells in those structures. RT-qPCR showed the mRNA level of differentiated lacrimal gland cell-related genes in the gland-like structures (**H**–**J**). Blue for DAPI. Bar represents 100 μm.

To test the differentiation potential of isolated progenitor cells, lacrimal gland epithelial cells from P1 to P10 were collected for 3-D organoid culture. Although a lower ratio of small gland-like structure formed from cells in higher passages, lacrimal gland epithelial cells in all test passages were able to form mini branching structures with similar morphology. Figure 8 shows representative data from P2 cells.

Lacrimal gland epithelial cells formed similar spheroids by using microtissue micro-molds (Fig. 8A). Several small buddings started to sprout out from lacrimal spheres cultured in the laminin gel on day 2 of culture (not shown), and gradually formed tubular and acinar-like structures in about 2 weeks of culture (Fig. 8B,C). Figure 8D and E illustrate K4 positive staining in tubular structure on day 5 sample and day 10 samples. The positive staining of lactoferrin (red) and AQP5 (green) was showed on the differentiation culture samples (Fig. 8F,G). Moreover, the real time PCR showed branching related gene *Sox9* (Fig. 8H) expression in early 3-D culture process and gradually increasing level of lacrimal gland differention markers during the 3-D culture (Fig. 8I–K).

## Discussion

The lacrimal gland is a compound tubuloacinar exocrine gland whose secretions account for the bulk of the aqueous portion of the tear film. Lacrimal gland progenitor cells are believed to play important roles in regeneration of damaged lacrimal glands and, therefore, suggested to have a therapeutic role in aqueous-deficient dry eye^3^. However, studies on lacrimal gland progenitor cells and gland regeneration capability are relatively limited, when compared to other similar glandular tissues^8, 25, 33^. Since the rabbit lacrimal gland is most similar to that of humans among the common models^34^, here we chose kind of adult rabbit as a model and first demonstrated that its lacrimal gland is capable of repair after main excretory duct ligation and reopening, which could induce lacrimal gland epithelial proliferation and reverse tear secretion damage after injury.

Duct ligation-induced injury was previously used to study regeneration in salivary glands, pancreas and liver. Several studies on exocrine glands, such as the pancreas, salivary, and mammary glands, showed that stem/progenitor cells exist in these tissues and are involved in their regeneration^4–7^. It was also reported that the murine lacrimal gland is capable of repair following experimentally induced injury by injection of IL-1 into the exorbital lacrimal glands^25^. In support of the notion of self regeneration capability in adult lacrimal gland, the main excretory duct ligation model might provide additional information of lacrimal gland repair without introducing external inflammatory factors.

Here, we report on the first ductal ligation study in rabbit lacrimal glands. Our results are consistent with those observed with a similar injury model in other gland tissues, including decreased gland weight, dysfunction of secretion, and proliferation of ductlike structures. As our goal was to provide evidence of the existence of lacrimal gland progenitor cells in adult tissue, we chose to use a surgical process allowing temporary main excretory duct ligation for 3 days, followed by reopening of the excretory duct and evaluation of the potential for self-repair. We noticed significant lacrimal gland epithelial cell proliferation after this short-term ductal ligation-induced injury and reversible tear production in around 10 days. The tear reduction level induced by ligature is also comparable to other studies with unilateral main lacrimal gland resection^35^. The disparity of gland weight regain and tear production recovery might indicate the compensation mechanism of lacrimal gland secretory cells. Long-term ligation might show different results during tissue repair.

Two possible mechanisms could be involved in lacrimal gland tissue repair and regeneration: expansion of stem/progenitor cells and/or trans-differentiation of existing cells. As we used the rabbit as an animal model, it is not applicable to do the lineage tracing experiment to identify the origin of regenerated cells in this species. However, we used several approaches to evaluate stem/progenitor cell-related marker expression, isolation and expansion of proliferative cells, and 3-D differentiation cultures. The results provided evidence to support the hypothesis that tissue-specific progenitor cells exist or, at least, develop after ligation injury. Further colocalization experiment on the very early time point would help to elucidate the participation of dedifferentiation process if existed.

The cell lineage of a tissue at a specific developmental stage provides important information for characterizing tissue-specific stem cells. During lacrimal gland development, a gland bud originates from conjunctival epithelium. The bud then branches and differentiates into mature tissue. This suggests that ocular surface epithelial cells and lacrimal gland cells may have similar specific stem/progenitor cell markers. Several papers suggested that K14, ∆Np63, and nestin are ocular surface stem/progenitor cell markers^36–39^. In the current study, temporarily ligated lacrimal gland tissues were found to have a significant increase in the level of expression of K14 and ∆Np63 compared to controls. As we expected, this correlated with higher mRNA and protein levels for these stem/progenitor cell-related genes, as assessed by RT-qPCR, immunostaining, and western blotting. This evidence points towards the existence of lacrimal gland progenitor cells after the injury.

In the current study, we also isolated and expanded lacrimal gland epithelial progenitor cells from injured glands during tissue repair. Those cells can be propagated from a single cell to form a monolayer and then passaged over 12 times. These cells are also positive for ocular surface stem/progenitor cell-related markers including K14, ABCG2, ∆Np63 and K15^40^.

However, using the current rabbit model, it is still not completely clear if these proliferating cells arise from resident stem/progenitor cells or if dedifferentiation of other cell types is responsible for the tissue regeneration. Double staining showed that only some of the ∆Np63 positive cells are K4 or SMA positive in ligation samples. It indicates multiple mechanisms involved in this regeneration process. Mice of a specific genetic background might be an alternative experimental animal model to address this question.

The EMT is known to play an important role in wound healing. There is evidence to suggest that differentiated epithelial cells that undergo EMT can gain stem cell-like properties^41^. Separately, there is evidence demonstrating the existence of stem/progenitor cells in mouse lacrimal glands that were injured by IL-1 injection. The process there also involved EMT process^42^. The current study suggests that the EMT may be involved in the activation of stem/progenitor cells in rabbit lacrimal glands after ligation-induced injury, with the elevation of EMT-associated gene expression in the proliferateing epithelial cells in ligated tissues. Moreover, ∆Np63 was reported to be the factor triggering EMT and conferring stem cell properties in normal human keratinocytes^43^, which is also consistent with our data. Those SMA positive cells might come from the EMT process, as well.

Multipotency is a major characteristic of adult stem cells and secretory cells–acinar cells compose the majority of cells and are the most important cell type in the functional lacrimal gland. Thus, we tested the differentiation potential of our lacrimal gland progenitor cells isolated from the ligated tissues in laminin gel 3-D cultures. As shown in Fig. 8, lacrimal gland progenitor cells formed acinotubular structures within 2 weeks. The branching related gene *Sox9* ^44^ was elevated in the early stage of 3-D culture. Meanwhile, there was positive staining and elevated mRNA level of ductal epithelial markers and secretory markers in those structures, indicating the direction of duct cell and acinar cell differentiation of our lacrimal gland epithelial progenitor cells.

During many biological processes, cell–cell contact through the formation of functional junctions or direct contact is important for communication with other cells. That contact allows cells to receive complex signals from their environment that can regulate development, homeostasis, and even disease progression. Epithelial-mesenchymal interactions are known to be extremely important for lacrimal gland development. Further studies, including mesenchymal composition in culture, should be done for organoid formation evaluation and later functional tests.

In summary, regeneration and EMT occur in temporarily ligated rabbit lacrimal glands. The proliferative lacrimal gland epithelial cells can be isolated and expanded from ligated tissue, and formed acinotubular structures in 3-D culture. This study has several limitations because of its use of rabbits, resulting from limited available antibodies and inaccessible genetic tracing experiments, and the EMT process need to be further studied. However, this model still offers a useful approach for studying self-repair of this tear-secreting gland, to further investigate the stem/progenitor cells of the adult lacrimal gland and the growth factors and signaling pathways involved in the regeneration process and stem cell behavioral regulation. Those information might help to better develope new strategies to promote stem cell-based treatment of clinical lacrimal gland dysfunction and aqueous tear-deficient dry eye.

## Methods

### Experimental Animals

Female New Zealand white rabbits (1.8–2.0 kg, 8–10 weeks) were used for this study. All of the procedures were performed in accordance with the ARVO Statement for the Use of Animals in Ophthalmic and Vision Research and were approved by the Johns Hopkins Animal Care and Use Committee. Rabbits were randomized into ligated and control groups. The main excretory duct of the right lacrimal gland was ligated for 3 days and then reopened. Lacrimal glands from ligated animals were assayed on days 0, 1, 3, 7, 10, 20 and 30 after the ligature was released. In each individual experiment, samples were obtained from at least three rabbits for each subgroup. Contralateral glands were not used as controls in this study as, in studies of salivary glands, it was previously noted that compensatory hyperplasia occurred in the remaining gland when the contralateral gland was extirpated^45, 46^ or ligated^46^.

### Schirmer I Test

The Schirmer I test (SIt; without anesthesia) was performed in the control and ligated groups before ligation, and every other day after reopening for 2 weeks. After the rabbits were held in a restraining device, standard Schirmer test filter paper strips were folded at the 5-mm notch and inserted into the lower lateral one-third of the conjunctival fornix for 5 minutes. The length of the moistened paper was then measured and recorded. The test was performed two times for each rabbit and the average score used for analysis.

### Cell Preparation

Rabbit lacrimal gland cells were isolated using a previously described protocol^32^. Excised lacrimal glands were digested with an enzyme cocktail of Hank’s balanced salt solution, ethylenediaminetetraacetic acid (EDTA), and a mixture of collagenase (350 U/ml), hyaluronidase (300 U/ml), and DNase (40000 U/ml), followed by incubation in a shaking water bath at 37 °C for 45 minutes. To minimize contamination of non-target cells, a 70-μm cell strainer and 5% Ficoll were used. Characteristics of the isolated cells were evaluated by real-time quantitative reverse transcription PCR (RT-qPCR). Isolated cells were cultured in Dulbecco’s Modified Eagle Medium: Nutrient Mixture F-12 (DMEM/F12) supplemented with 1% insulin-transferrin-sodium selenite (ITS), 0.1% cholera toxin, 5% KnockOut Serum Replacement, 10 ng/ml epidermal growth factor (EGF), and 5 μM Y-27632 rho associated coiled-coil protein kinase (ROCK) inhibitor.

### Single Cell Assay

A single cell assay was performed on passage 1 (P1) cells to identify high proliferation capability. Monolayer cells in P0 were dissociated with Accutase^®^ and diluted to 10 cells/mL, and then 100 µL cell suspension was transferred to each well of 96-well plates. Cell density and cell growth was determined by using phase-contrast microscopy. Wells containing a single cell were used for study and cultured until they became confluent.

### Immunofluorescence Staining

For immunofluorescence analysis, 8 μm lacrimal gland sections were prepared. Samples were immunostained for stem/progenitor cell-related markers, including nestin, cytokeratin 14 (K14) and ΔNp63, epithelial cell marker pan-cytokeratin (PCK), duct cell-specific marker cytokeratin 4 (K4), myoepithelial marker alpha-smooth muscle actin (SMA) and EMT related marker Vimentin. To determine whether lacrimal gland progenitor cells underwent differentiation, immunostaining for lactoferrin and AQP5 were performed. The fluorescent dyes Alexa Fluor^®^ (AF) 488- or AF568-conjugated secondary antibodies were used. Nuclear staining was done with VECTASHIELD Mounting Medium with DAPI. Samples were analyzed under a Zeiss LSM 510 confocal microscope.

### RT-qPCR

Total RNA extraction and reverse transcription, the RNeasy Mini Kit and SuperScript III First-Strand Synthesis System, were used according to the manufacturers’ instructions. One microgram of purified total RNA was used for cDNA synthesis, followed by RT-qPCR amplification in a StepOnePlus Real-Time PCR System with a Power SYBR^®^ Green PCR Master Mix. Target primers specific to *K14, NES, P63, ABCG2, CDX2, PCNA, BMA1, CDH1, SNAL2, VIM, SOX9* and the primer for reference gene β-actin were designed using Primer 3 software.

### Western Blotting

Aliquots of 20 µg protein prepared from lacrimal gland tissues were resolved by 15% sodium dodecyl sulfate–polyacrylamide gel electrophoresis conducted with a Mini-PROTEAN II Electrophoresis apparatus. After electrophoresis, the separated proteins were transferred onto a nitrocellulose membrane by means of a Mini Trans-Blot^®^ apparatus following standard procedures. Nitrocellulose membranes were blocked at room temperature for 2 h with 5% skim milk and then incubated overnight at 4°c with 1000 times-diluted anti-K14/β-Tubulin antibodies. Membranes were washed and subsequently incubated for 1 h at room temperature with 5000 times-diluted HRP-conjugated anti-mouse IgG. Finally, they were subjected to the enhanced chemiluminescence (ECL) reaction followed by exposure to an X-ray film for the appropriate time.

### Three-Dimensional Culture

We used a spheroid culture method modified from one for neural stem cells, and then a floating organoid culture method modified from one used for thymic epithelial cells and lacrimal gland epithelium^34, 35^. Briefly, after isolation, lacrimal gland progenitor cells were resuspended in culture medium as 8 × 10^6^ cells/ml, and 50 μl of cell suspension was seeded in each micro-mold (Microtissues; http://www.microtissues.com). The formed spheroids were cultured in nonadhesion flasks for one more day, and then seeded into a 15 μl drop of laminin (6 mg/ml; Trevigen) sitting on a polycarbonate filter floating in serum-free medium for 2 weeks.

### Quantification of Immunofluorescence Staining and Western Blot

Images were processed in ImageJ software for data analysis. Signals were determined by following ImageJ user’s guide with at least 3 complicates in each subgroup. Data were exported to Prism 6 to generate the plot. Data are presented as staining area over nuclear number for K14, positive cell ratio for ΔNp63, and change in fold in protein expression after normalized to loading controls.

### Statistical Analysis

Results were expressed as means ± SEM (Standard Error of the Mean), and were statistically compared by ANOVA via SPSS software; p < 0.05 was considered statistically significant.

## Electronic supplementary material

Supplementary
