# Supplementary material for: Lacrimal Gland Repair after Short-term Obstruction of Excretory Duct in Rabbits
Source: Sci Rep. 2017 Aug 15;7:8290. doi: 10.1038/s41598-017-08197-2 (PMC5557958; doi:10.1038/s41598-017-08197-2)

Lacrimal Gland Regeneration after Short-term Obstruction of Excretory Duct in Rabbits

Hui Lin, MD, PhD,[1] Ying Liu, MD, PhD[1], Hong He, MD, PhD,[1] Benjamin Botsford, MD,[2] Samuel Yiu*, MD, PhD [1]

1. Wilmer Eye Institute, School of Medicine, Johns Hopkins University, Baltimore, MD, United States.
2. Tufts University School of Medicine, Boston, MA, United States.

*Corresponding author: Samuel Yiu, MD, PhD

400 N. Broadway, Baltimore, Rm 6041, MD 21231, United States. Tel.: +1-443-287-4890.

syiu2@jhmi.edu

Supplementary Figure 1: Western Blot for K14. The lanes from left to right represent control, D0, D1, D3, D7, D10, D20 and D30 samples representatively.

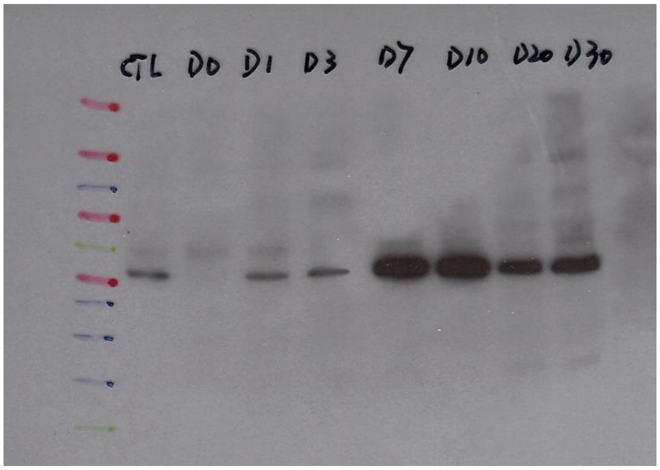

Supplementary Figure 2: Western Blot for T-tubulin. The lanes from left to right represent control, D0, D1, D3, D7, D10, D20 and D30 samples representatively.

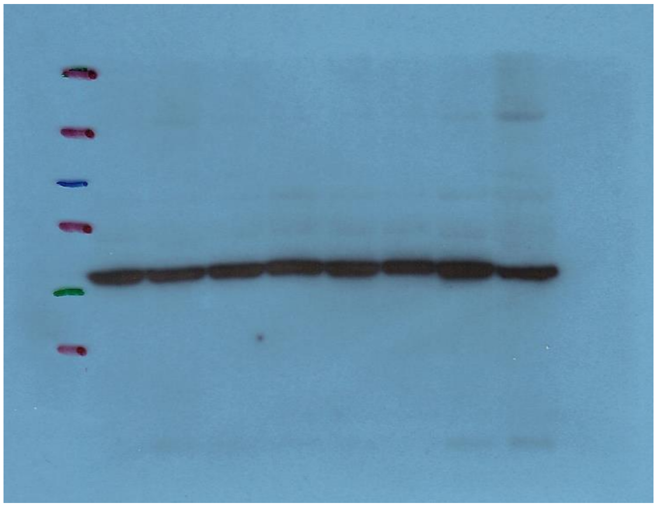

Supplement: Supplementary file 1 — Supplementary [file 41598_2017_8197_MOESM1_ESM.pdf]
